# Social support and its associated factors among older adult patients with intestinal stomas: a cross-sectional study in Guangxi, China

**DOI:** 10.3389/fpubh.2025.1630719

**Published:** 2025-09-05

**Authors:** Lijiao Wei, Ruili Wei, Fang Huang, Junwen Shen, Hongjun Li, Chanchan Li

**Affiliations:** Department of Colorectal and Anal Surgery, The First Affiliated Hospital of Guangxi Medical University, Nanning, China

**Keywords:** older adult patients with intestinal stoma, social support, influencing factors, Guangxi, multiple linear regression analysis, stoma-related complications

## Abstract

**Background:**

Social support plays a crucial role in the rehabilitation and psychosocial well-being of older adult individuals following intestinal stoma surgery. However, regional disparities and influencing factors of social support in this population remain insufficiently studied, particularly in underserved areas of China. This study aimed to assess the current level of social support and identify its influencing factors among older adult patients with intestinal stomas in the Guangxi region of China.

**Methods:**

A cross-sectional study was conducted from September to December 2024 involving 162 older adult patients (aged ≥60) who underwent intestinal stoma surgery within the previous year. Participants were recruited from stoma outpatient clinics in three tertiary hospitals in Guangxi. Data were collected using a self-designed demographic questionnaire and the validated Social Support Rating Scale (SSRS). Descriptive statistics, t-tests, ANOVA, and multiple linear regression analyses were performed using SPSS 26.0 to identify significant predictors of social support.

**Results:**

The overall mean SSRS score was 39.12 ± 4.57, indicating a moderate level of social support. Objective support (19.81 ± 2.54) scored the highest among the three dimensions, followed by subjective support (11.45 ± 1.99) and utilization of support (7.86 ± 1.39). Multivariate analysis identified the type of primary caregiver and the presence of peristomal skin complications as independent predictors of social support (*p <* 0.05). Other significantly associated factors included age, education level, and the patients’ and their families’ acceptance of the stoma.

**Conclusion:**

Older adult patients with intestinal stomas in Guangxi experience moderate social support, influenced by both clinical and psychosocial factors. Targeted interventions that improve caregiver engagement and manage stoma-related complications are essential for strengthening social support systems and enhancing post-surgical outcomes in this vulnerable population.

## Introduction

With the ongoing acceleration of population aging in China, the number of older adult patients undergoing surgical treatments is steadily increasing. Among these, intestinal stoma surgery has become a key intervention for conditions such as colorectal cancer, intestinal obstruction, and other gastrointestinal diseases. Postoperative recovery not only involves the restoration of physiological function but also requires significant psychosocial adjustment (1). For older adult patients, the presence of a stoma can lead to lowered self-esteem, social isolation, and diminished life satisfaction (2). Therefore, effective psychosocial support has become a critical component of clinical management (3). An increasing body of evidence indicates that social support plays a vital role in recovery outcomes for stoma patients, closely influencing their quality of life, mental health, and complication rates (4–6). Nevertheless, existing research largely emphasizes nursing care techniques, with insufficient attention to the structural aspects of social support—especially in older adult populations and those residing in remote areas. This gap highlights the need for integrated theoretical and empirical research to comprehensively assess the role of social support in the postoperative recovery of older adult intestinal stoma patients.

As a region with a high concentration of ethnic minorities, Guangxi presents a complex sociocultural structure and uneven distribution of healthcare resources, making it a representative area for studying regional health issues among older adults. Older adult patients with intestinal stomas in this region face numerous challenges during postoperative recovery, including limited access to medical services, low health literacy, and deeply rooted traditional beliefs in bodily integrity (7). Preliminary investigations reveal that the follow-up compliance rate among stoma patients in the Guangxi region is significantly lower than the national average, while the incidence of postoperative complications is markedly higher (8). These findings suggest that there are substantial barriers to obtaining adequate social support within this population. Importantly, most existing studies have relied on subjective descriptions or assessed social support using a single-dimensional approach, lacking a comprehensive analysis of its three core dimensions—objective support, subjective support, and support utilization (9). In the context of stoma care, a critical gap remains in the scientific quantification of these support dimensions and in understanding their interrelationships. Furthermore, despite the high clinical relevance and modifiability of peristomal skin complications, current research has yet to consistently understand their association with social support.

Theoretically, research on social support should be grounded in well-established conceptual models for systematic analysis. According to Social Support Theory, an ideal social support system comprises three key dimensions: objective support (e.g., financial assistance and tangible help), subjective support (the individual’s perceived emotional care), and support utilization (the willingness to seek help and the ability to access resources) (10–12). A lack of social support or inadequate utilization of available support can directly impair a patient’s confidence in recovery and adherence to treatment (13, 14). Within this framework, family-centered care plays a critical moderating role, particularly in relation to the type and quality of the primary caregiver. Different caregivers—such as spouses, children, other relatives, or healthcare professionals—may offer varying forms, frequencies, and perceived support effectiveness (13–15). Patients tend to rely more heavily on family-based caregiving systems in regions like Guangxi, where healthcare services are relatively underdeveloped. As a result, the family support structure becomes a major determinant of social support levels. Moreover, postoperative factors such as peristomal skin complications not only increase the complexity of caregiving but may also exert psychological burdens on patients, thereby reducing their motivation and capacity to seek and utilize social support (16–18). Consequently, it is essential to explore the structure and functional pathways of social support systems through both theoretical and empirical lenses.

Based on this background, this study aimed to systematically assess the level of social support and identify its primary influencing factors among older adult patients with intestinal stomas in the Guangxi region, using the validated Social Support Rating Scale (SSRS). Specifically, the study examined: (1) variations in social support scores across demographic variables (e.g., age, gender, and education level); (2) the effects of clinical factors, including peristomal skin complications and stoma type; and (3) the moderating role of the primary caregiver within the family support structure. This research offers novel insights by focusing on an economically underdeveloped region, thereby addressing a key gap in the existing literature. It further explores the interaction between clinical complications and psychosocial factors, highlighting the significance of integrated family and community-based care. The findings are expected to inform the development of targeted support strategies and improve long-term outcomes in this vulnerable population.

## Materials and methods

### Participants

**Figure 1:**
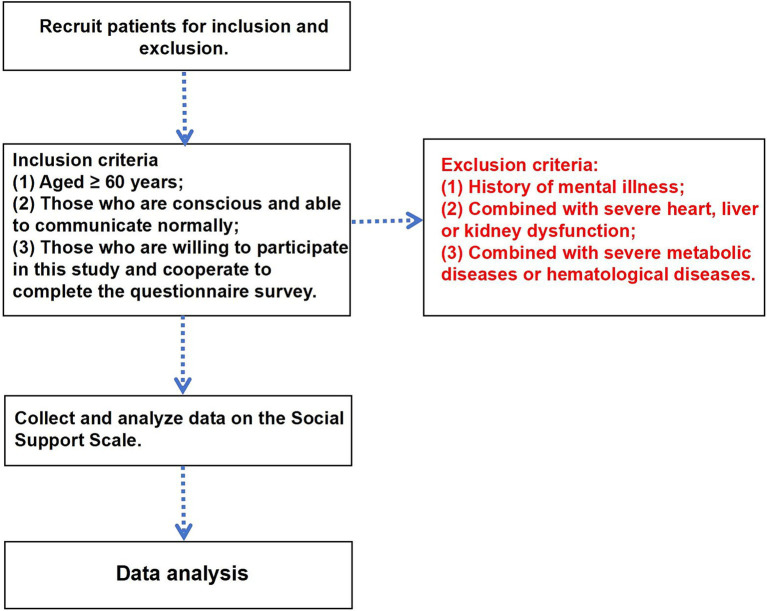
Flowchart of the experimental research design.

This study adopts a cross-sectional design and includes 162 older adult patients who underwent intestinal stoma surgery within the past year and returned for follow-up or consultation at stoma outpatient clinics in three tertiary hospitals in the Guangxi region between September and December 2023 (Figure 1). This study was conducted by the ethical standards of the institutional and/or national research committee and with the 1964 Helsinki declaration and its later amendments or comparable ethical standards. The research protocol was reviewed and approved by the Clinical Ethics Committee of the First Affiliated Hospital of Guangxi Medical University. Informed consent was obtained from all individual participants included in the study or their legal guardians.

Inclusion criteria were: (1) age ≥ 60 years; (2) clear consciousness and the ability to communicate effectively; and (3) willingness to participate in the study and complete the questionnaire. Exclusion criteria included: (1) a history of psychiatric disorders; (2) severe dysfunction of the heart, liver, or kidneys; and (3) complicated with severe metabolic disorders or hematologic diseases.

### General information questionnaire

The research team independently developed the general information questionnaire based on a review of relevant literature. It includes variables such as gender, age, educational level, monthly household income per capita, method of payment for medical expenses, relationship with family members, and the primary caregiver after hospital discharge.

### Social support scale

The SSRS is used to assess participants’ social support and includes 10 items across three dimensions: objective support (Items 2, 6, 7), subjective support (Items 1, 3, 4, 5), and support utilization (Items 8, 9, 10). The SSRS (Table 1) responses are measured using a 4-point Likert scale (ranging from 1 to 4), except for Items 5 to 7, which are scored based on the number of applicable options selected. The total score is the sum of all three dimensions, ranging from 8 to 50 points. Scores < 20 indicate very poor support, 20–30 poor support, 30–40 moderate support, and > 40 good support. The three dimensions of the scale account for 55.84% of the total variance. The scale’s overall Cronbach’s alpha coefficient is 0.78, indicating good reliability and structural stability.

**Table 1:** Items and scoring methods of the social support rating scale (SSRS).

Dimension	Item no.	Item content	Scoring method
Objective support	2	In emergency situations, the sources from which you have received financial support or practical help include:	1 point per selected source (max 4 points)
	6	In the past year, your level of interaction with non-relative members such as colleagues/friends/neighbors:	1 = Very little, 2 = Occasionally, 3 = Frequently, 4 = Very frequently
	7	In the past year, the number of times you participated in activities of social organizations (e.g., unions, religious groups, etc.):	1 = Never, 2 = 1–2 times, 3 = 3–5 times, 4 = ≥6 times
Subjective support	1	How many close friends do you have who can provide support and help?	1 = None, 2 = 1–2, 3 = 3–5, 4 = ≥6
	3	The level of mutual assistance between you and your neighbors:	1 = None, 2 = Occasionally, 3 = Moderate, 4 = Frequently
	4	The level of mutual assistance between you and your colleagues:	1 = None, 2 = Occasionally, 3 = Moderate, 4 = Frequently
	5	The level of support and care you receive from family members (e.g., spouse/children):	1 = None, 2 = Minimal, 3 = Moderate, 4 = Full support
Support utilization	8	How you confide when facing troubles:	1 = Never confide, 2 = Only to acquaintances, 3 = Professional consultation, 4 = Actively seek multiple help
	9	How you seek help when facing difficulties:	1 = Handle alone, 2 = Seek help from relatives/friends, 3 = Seek help from organizations, 4 = Comprehensive seeking
	10	Your initiative in participating in group activities (e.g., community/patient groups):	1 = Never participate, 2 = Passively participate, 3 = Actively participate, 4 = Organize and plan

### Data collection procedure

During the patients’ visits to the stoma clinic, outpatient nurses collected data through questionnaire surveys. The specific questionnaire content is shown in the General Information Survey Form. Before data collection, the researchers conducted unified training for the outpatient nurses. Before the survey, the outpatient nurses obtained informed consent from patients and had them sign the corresponding forms. The nurses explained the purpose, significance, and completion method of the survey to patients face-to-face. The completion time was approximately 30 min. The outpatient nurses read each item in non-suggestive language for patients with reading difficulties or visual impairments. After the patients understood the questions, they provided answers, which the outpatient nurses recorded.

### Outcome measures

The primary outcome of this study is the level of social support among older adult patients with intestinal stomas. Social support is assessed using the SSRS, as detailed in Table 1. The scale comprises three dimensions: objective support, subjective support, and support utilization.

### Statistical analysis

All data were analyzed using SPSS version 26.0. Continuous variables were expressed as mean ± standard deviation (*x̄* ± *s*) and compared using independent-sample t-tests or one-way ANOVA. Categorical variables were presented as frequencies and percentages [n (%)] and compared using the chi-square test. To identify factors associated with social support levels, univariate analyses were first conducted to screen candidate variables. Variables with statistical significance were tested for multicollinearity using the variance inflation factor (VIF < 5), and eligible predictors were entered into multiple linear regression models. In addition to modeling total social support scores, separate regression models were constructed for the three SSRS subdimensions, objective support, subjective support, and support utilization, using the same six predictors: age, education level, self-acceptance of the stoma, family acceptance of the stoma, primary caregiver, and presence of peristomal skin complications. Stepwise regression was applied, and regression coefficients and model fit indices (*B* values, *p*-values, and *R*^2^) were reported. A two-tailed *p*-value < 0.05 was considered statistically significant.

## Results

### Overall social support score

**Figure 2:**
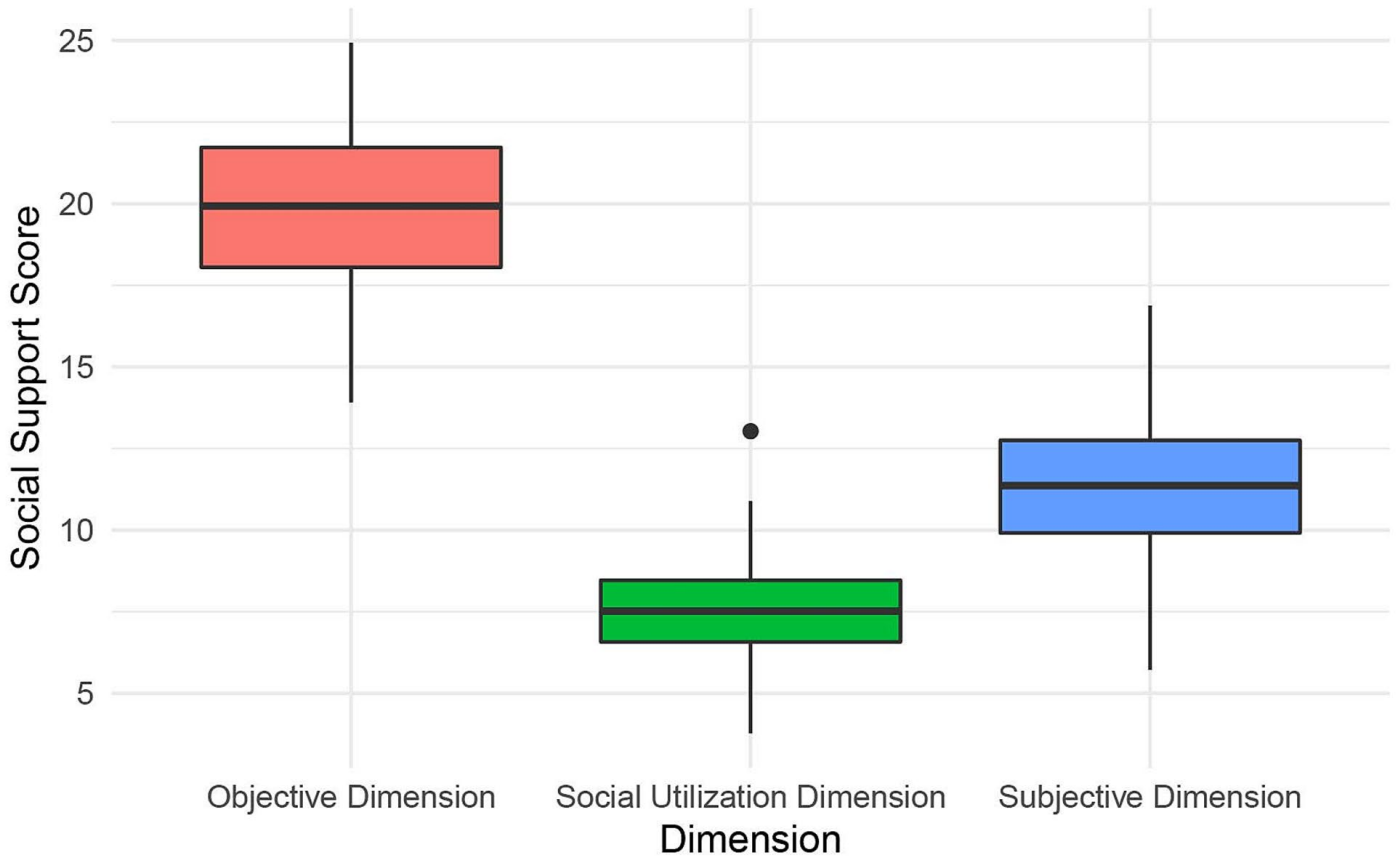
Overall social support scores among older adult patients with intestinal stomas in the Guangxi Region.

The overall social support score among older adult patients with intestinal stomas in the Guangxi region is 39.12 ± 4.57. Specifically (Figure 2 and Table 2), the score for objective support (19.81 ± 2.54) is significantly higher than those for subjective support (11.45 ± 1.99) and support utilization (7.86 ± 1.39). These findings reflected relatively higher levels of tangible support, while scores related to perceived emotional support and the use of support resources were lower.

**Table 2:** Total social support scores (*n* = 162, *X* ± s).

Item	Number of items	Minimum score	Maximum score	Mean ± Standard deviation	Dimension mean score	Rank
Subjective dimension	4	5	17	11.45 ± 1.99	2.86 ± 0.49	2
Objective dimension	3	8	26	19.81 ± 2.54	6.60 ± 0.84	1
Social utilization dimension	3	3	11	7.86 ± 1.39	2.62 ± 0.46	3
Total social support score	10	16	51	39.12 ± 4.57	–	–

### Item-level social support scores

**Figure 3:**
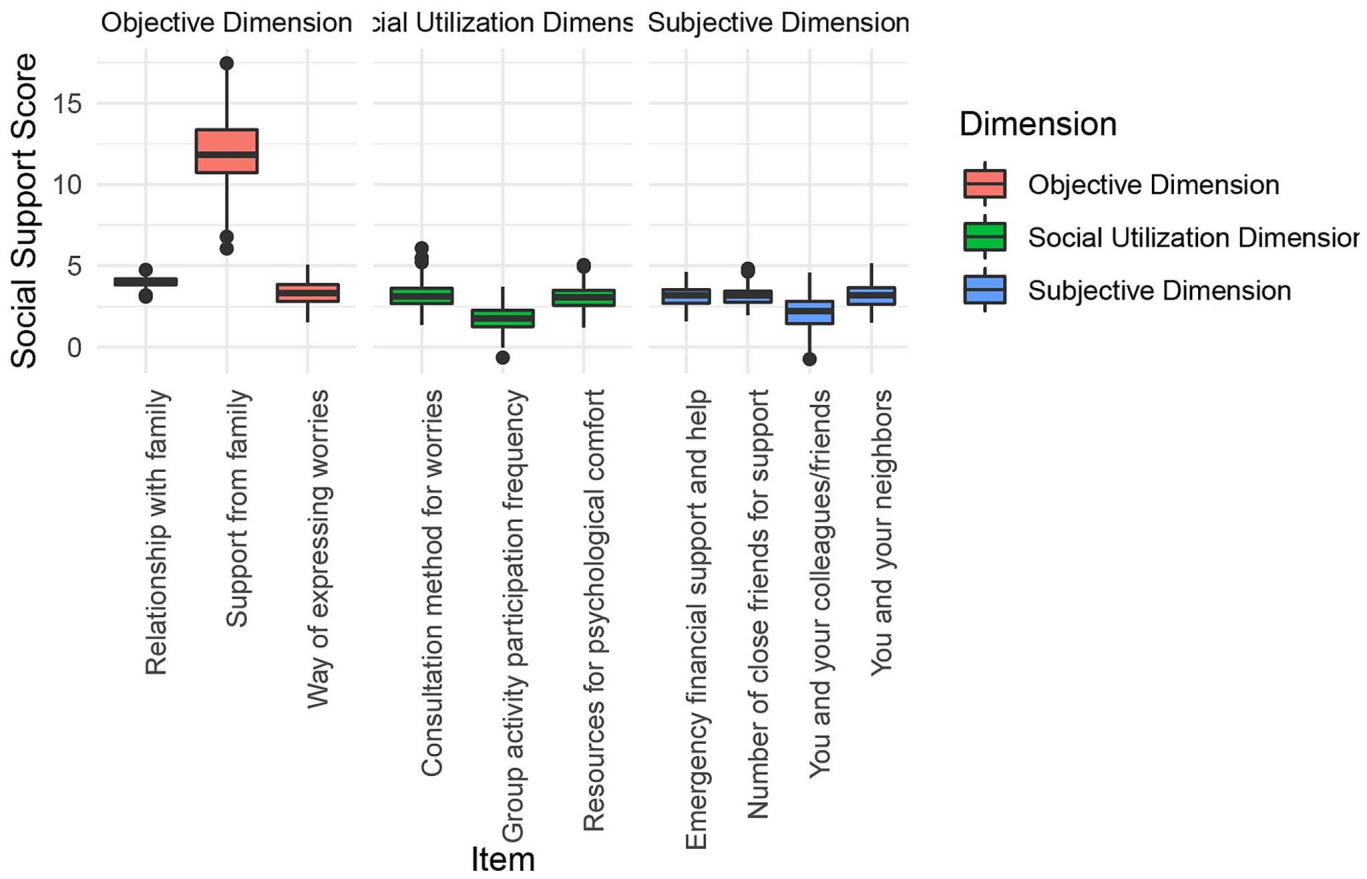
Item-level scores of the social support scale.

This study analyzed item-level social support among older adult patients with intestinal stomas in the Guangxi region (Table 3 and Figure 3). In the subjective support dimension, the highest-scoring item was “Your relationship with your neighbors” (3.14 ± 0.81). In the objective support dimension, the item “Support and care received from family members” had the highest score (12.52 ± 2.25). For the support utilization dimension, the highest score was recorded for “Access to resources that provide psychological comfort and care” (3.09 ± 0.69). Although this item scored relatively higher within its dimension, the overall score remained lower compared to the other two dimensions, suggesting limited engagement with psychological support resources.

**Table 3:** Comparison of scores for each item of social support (*n* = 162, *X* ± *s*).

Dimension	Item	Minimum score	Maximum score	Score	Rank
Subjective dimension	3. Your relationship with neighbors	1	4	3.14 ± 0.81	1
	4. Your relationship with colleagues and friends	1	4	2.15 ± 1.00	4
	5. In the past, when you encountered emergencies, the sources of financial support and practical help you received	1	5	3.09 ± 0.60	2
	1. Number of close friends you can rely on for support and help	2	4	3.07 ± 0.50	3
Objective dimension	2. Your relationship with family members	1	4	3.96 ± 0.33	2
	6. How you express your worries	1	4	3.32 ± 0.78	3
	7. Amount of support and care received from family members	5	20	12.52 ± 2.25	1
Social utilization dimension	8. Resources for psychological comfort and care you received (during emergencies)	1	6	3.09 ± 0.69	1
	9. How you seek advice when encountering troubles	1	4	3.08 ± 0.79	2
	10. Frequency of participation in group or organizational activities	1	4	1.70 ± 0.74	3

### Univariate analysis of the influence of demographic characteristics on social support in older adult patients with intestinal stoma

**Figure 4:**
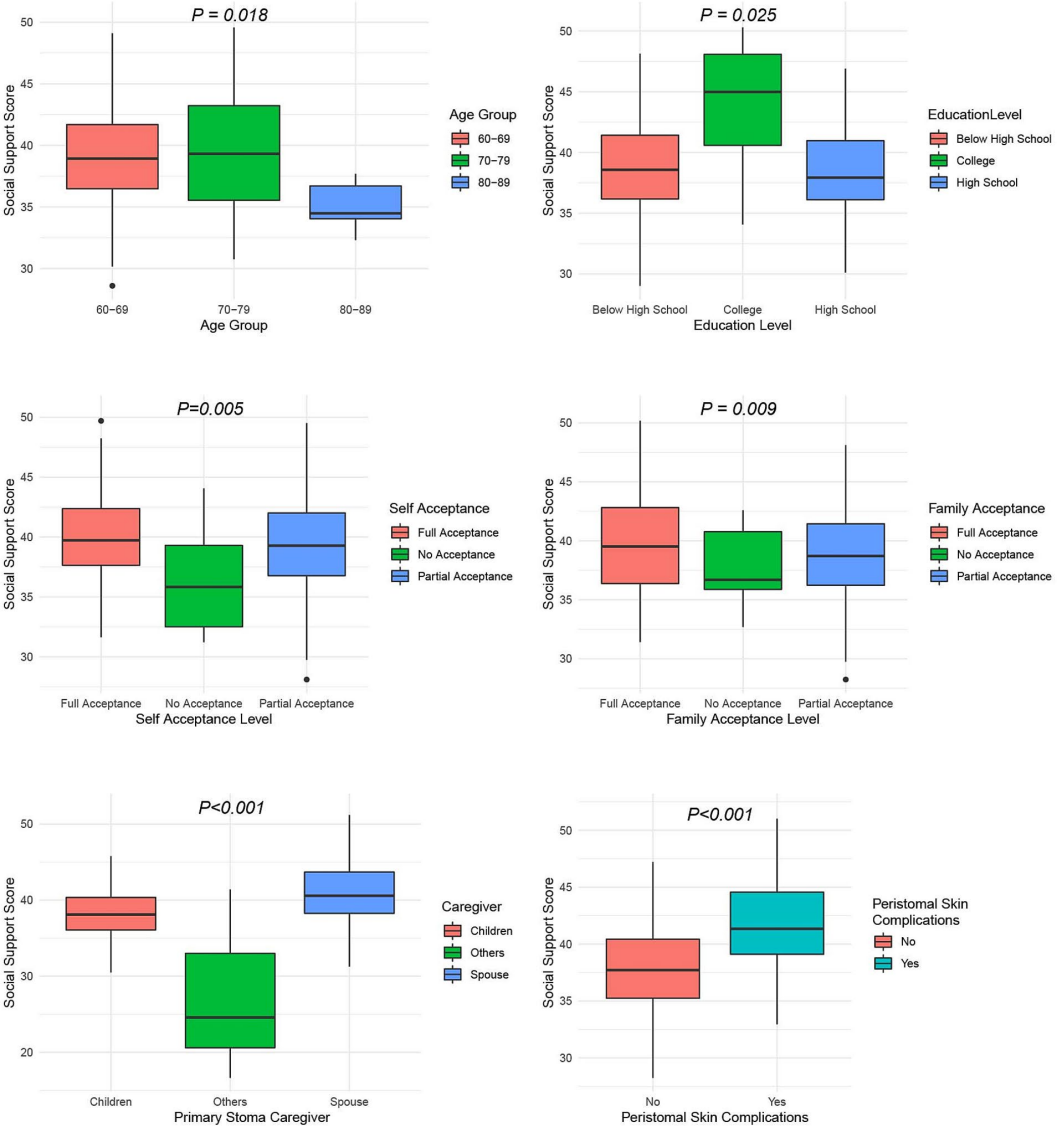
Univariate analysis of demographic characteristics and social support among older adults patients with intestinal stomas.

The univariate analysis results (Table 4) show that several demographic characteristics significantly influence the total social support score among older adult patients with intestinal stomas (*p <* 0.05, Figure 4). These include age (*p =* 0.018), educational level (*p =* 0.025), the patient’s acceptance of the stoma (*p =* 0.005), family members’ acceptance of the stoma (*p =* 0.009), the identity of the primary stoma caregiver (*p <* 0.001), and the presence of peristomal skin complications (*p <* 0.001) are all statistically significant factors. In contrast, variables such as gender, method of medical expense payment, and household income do not show statistically significant associations with social support (*p >* 0.05).

**Table 4:** Comparison of total social support scores by demographic characteristics (*n* = 162, *X* ± *s*).

Item	Group	Number of cases	Total social support score	*T*/*F* value	*P*-value
Gender	Male	114	38.82 ± 4.31	−1.234	0.221
	Female	48	39.85 ± 5.11		
Age	60 ~ 69 years	120	39.13 ± 4.56	4.116	0.018
	70 ~ 79 years	34	40.06 ± 4.53		
	80 ~ 89 years	8	35.00 ± 2.56		
Medical payment method	Medical Insurance	5	39.30 ± 6.54	1.211	0.301
	Self-pay	16	37.44 ± 4.53		
Monthly household income	≤2000 RMB	112	38.79 ± 3.68	1.104	0.334
	2001 ~ 4,000 RMB	41	40.02 ± 6.20		
	≥4,001 RMB	9	39.22 ± 5.82		
Education level	Junior High School or Below	129	38.91 ± 4.47	3.764	0.025
	High School	25	38.84 ± 4.25		
	College or Above	8	43.38 ± 5.52		
Family relationship	Good	126	39.45 ± 4.70	1.723	0.087
	Fair	36	37.97 ± 3.91		
Stoma duration	0 ~ 3 months	91	39.03 ± 4.48	0.312	0.732
	4 ~ 6 months	57	39.44 ± 4.26		
	7 ~ 12 months	14	38.43 ± 6.37		
Stoma type	Temporary	141	38.00 ± 4.78	−1.161	0.256
	Permanent	21	39.29 ± 4.53		
Stoma location	Ileostomy	36	38.00 ± 4.12	−1.798	0.077
	Colostomy	126	39.44 ± 4.65		
Self-acceptance of stoma	Partial Acceptance	88	39.14 ± 4.78	5.538	0.005
	Full Acceptance	48	40.38 ± 4.26		
	No Acceptance	26	36.77 ± 3.47		
Family acceptance of stoma	Partial Acceptance	102	38.44 ± 4.42	4.845	0.009
	Full Acceptance	52	40.69 ± 4.52		
	No Acceptance	8	37.63 ± 4.71		
Primary stoma caregiver	Spouse	64	40.75 ± 4.82	14.982	<0.001
	Children	95	38.35 ± 3.40		
	Others	3	29.00 ± 11.79		
Stoma complications	Yes	17	39.53 ± 4.70	0.386	0.700
	No	145	39.08 ± 4.57		
Peristomal skin complications	Yes	52	41.54 ± 4.37	4.946	<0.001
	No	110	37.98 ± 4.22		

### Multivariate analysis of factors influencing social support in older adult patients with intestinal stoma

**Figure 5:**
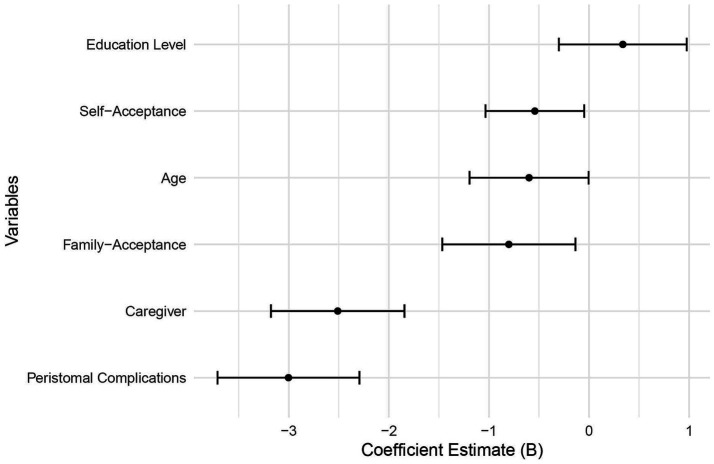
Multivariate analysis of factors influencing social support in older adult patients with intestinal stomas.

Multiple linear regression was performed with total social support score as the dependent variable and age, educational level, patient acceptance of the stoma, family acceptance of the stoma, primary stoma caregiver, and presence of peristomal skin complications as independent variables (Table 5). The analysis showed that primary stoma caregiver (*p <* 0.05) and peristomal skin complications (*p <* 0.05) were independently associated with social support scores (Table 6 and Figure 5). Patients whose primary caregiver was a spouse had higher social support scores, whereas those with peristomal skin complications had lower scores. Age (*p =* 0.316), educational level (*p =* 0.598), patient acceptance of the stoma (*p =* 0.275), and family acceptance of the stoma (*p =* 0.230) were not statistically significant. The model explained 20.4% of the variance in social support scores (*R*^2^ = 0.204). Although the model’s explanatory power was limited, the significant factors (primary stoma caregiver and peristomal skin complications) provided important insights for improving patients’ social support levels. Variance inflation factors were all below 5, and residual diagnostics indicated that the assumptions of normality, homoscedasticity, and independence were met.

**Table 5:** Assignment description of social support influencing factors.

Factor	Variable name	Assignment description
Age	X1	60 ~ 69 years = 1, 70 ~ 79 years = 2, 80 ~ 89 years = 3
Education level	X2	Junior High School or Below = 1, High School = 2, College or Above = 3
Self-acceptance of stoma	X3	Full Acceptance = 1, Partial Acceptance = 2, No Acceptance = 3
Family acceptance of stoma	X4	Full Acceptance = 1, Partial Acceptance = 2, No Acceptance = 3
Primary stoma caregiver	X5	Spouse = 1, Children = 2, Others = 3
Peristomal skin complications	X6	Yes = 1, No = 2
Total social support score	Y	Raw Data

**Table 6:** Independent variables entering the regression equation for social support influencing factors and their related parameters.

Variable	*B*	Sb	Beta	*T*	*P*
Constant	50.693	2.218	-	22.852	0.000
Age	−0.599	0.595	−0.071	−1.007	0.316
Education level	0.338	0.639	0.039	0.529	0.598
Self-acceptance of stoma	−0.541	0.493	−0.086	−1.096	0.275
Family acceptance of stoma	−0.801	0.665	−0.095	−1.204	0.230
Primary stoma caregiver	−2.511	0.667	−0.280	−3.763	<0.001
Peristomal skin complications	−3.003	0.709	−0.305	−4.235	<0.001

Three separate multiple linear regression models were constructed for the subdimensions of social support. The results indicated that both the primary caregiver and peristomal skin complications were significant predictors across all dimensions, with particularly strong effects observed in objective support (*B* = −2.743, −3.110; *p <* 0.001) and support utilization (*B* = −1.145, −1.379; *p <* 0.01). The model *R*^2^ values were 0.302 (objective support), 0.189 (subjective support), and 0.276 (support utilization), all higher than that of the total social support score model (*R*^2^ = 0.204), suggesting that dimension-specific modeling provides greater explanatory power (Table 7).

**Table 7:** Multiple linear regression models for three dimensions of social support (*n* = 162).

Variable	B (Objective)	*p* (Objective)	B (Subjective)	*p* (Subjective)	B (Utilization)	*p* (Utilization)
Age	−0.482	0.036	−0.231	0.248	−0.338	0.118
Education level	0.452	0.041	0.085	0.736	0.107	0.648
Self-acceptance of stoma	−0.498	0.027	−0.291	0.164	−0.255	0.201
Family acceptance of stoma	−0.775	0.030	−0.429	0.198	−0.388	0.172
Primary caregiver	−2.743	<0.001	−1.621	0.009	−1.145	0.004
Peristomal skin complications	−3.110	<0.001	−1.458	0.011	−1.379	0.001
*R* ^2^	0.302		0.189		0.276	

B: unstandardized regression coefficient; *p* < 0.05 indicates statistical significance. *R*^2^: coefficient of determination.

## Discussion

This cross-sectional study surveyed 162 older adult intestinal stoma patients in Guangxi and found that their overall social support score was moderate (39.12 ± 4.57), yet significantly lower than those reported in similar populations across other regions of China. This indicates a regional gap in psychosocial support that warrants attention. Multiple linear regression identified the type of primary caregiver and the presence of peristomal skin complications as independent predictors of social support. These findings highlight structural challenges in care for older adults stoma patients in under-resourced areas and provide an evidence base for developing targeted, region-specific interventions.

This study found that the overall level of social support among patients was lower than that of stoma patients in eastern China, consistent with previous findings that patients in economically underdeveloped regions experience significant disadvantages in terms of social support (19). Similar to earlier research (20), this study further confirmed that patients living in rural areas had lower social support scores due to disadvantages in transportation, economy, and access to medical resources. In addition, this study found that having a spouse or children as the primary stoma caregiver significantly improved patients’ social support scores, aligning with prior studies on the importance of family support among patients with chronic diseases (21). Notably, this study also revealed the impact of ethnic and cultural differences on social support levels—patients from ethnic minority groups such as the Zhuang had significantly lower scores than Han patients, consistent with existing theories on the influence of cultural beliefs, highlighting the importance of culturally sensitive interventions in border regions (22).

This study found that peristomal skin complications not only affect patients’ physical health but may also indirectly reduce perceived social support by lowering self-efficacy and limiting social interactions. This suggests a potential bidirectional interaction between physiological burden and psychosocial adaptation, extending the traditional unidirectional perspective that “social support improves health outcomes.” Peristomal complications increase emotional distress and social restriction, negatively impacting quality of life—yet their psychological and social consequences have long been underrecognized (23). Prior studies have shown that unresolved skin issues can impair social functioning and support systems and are significantly associated with psychological well-being (24, 25). Furthermore, a lack of nursing guidance may further weaken patients’ self-management confidence and social connectedness (26). These findings suggest that peristomal complications should be viewed not only as clinical problems but also as critical psychosocial variables influencing social support.

In addition to macro-level factors such as care infrastructure and resource accessibility, this study also highlights the role of individual psychological and behavioral mechanisms in shaping social support. Some patients experienced stigma related to body image changes caused by the stoma, leading to social withdrawal and dependency, which reduced their initiative in expressing support needs and seeking help (27). Social withdrawal has been shown to be negatively associated with perceived support, especially among patients with permanent stomas (28). Our findings further revealed that patients with stoma-related complications had lower social support scores, indicating that physical discomfort may reduce self-efficacy and willingness to engage socially, creating a negative cycle of “physical burden – psychological response – decline in social support” (29). Self-efficacy is believed to moderate the relationship between social support and health outcomes and is closely related to factors such as educational level and family acceptance of the stoma (30). Positive psychological interventions, such as hope training and resilience development, have been shown to enhance psychological capital and improve both life satisfaction and support utilization (31). Taken together, these findings support a bidirectional relationship between social support and psychosocial health, in line with the biopsychosocial model, and provide theoretical guidance for the development of more targeted and individualized care strategies. Future research could employ path or mediation analysis to validate the proposed mechanism and inform intervention design.

This study offers practical implications for clinical and policy development. It is recommended that primary healthcare centers establish an integrated support system linking patients, families, volunteers, and providers, drawing on successful community-based models (32). For rural patients, a combination of remote stoma care and home visits may improve accessibility. Additionally, health insurance policies should cover stoma care supplies and include targeted subsidies under rural health assistance programs to reduce the financial burden and improve social support.

Despite revealing the current status and influencing factors of social support among older adult patients with intestinal stomas in Guangxi, this study has several limitations. First, the sample was mainly drawn from economically underdeveloped areas, leading to the underrepresentation of high-income and highly educated groups. This may have affected the accuracy of estimates for these populations and limited the generalizability of related conclusions. Future studies should expand the sample size and include provincial-level hospitals to improve the socioeconomic balance. Second, the study lacked adequate age stratification, making it difficult to capture heterogeneity within older adult populations. More refined stratification methods are recommended to enhance representativeness and statistical power. Third, the cross-sectional design did not allow for assessment of temporal changes in social support or causal inference. Longitudinal studies with improved measurement tools are needed. Fourth, qualitative variables were entered directly into the regression model, which may have overlooked potential non-linear effects. Future studies should incorporate dummy variables or use ANOVA in larger samples for further validation. Fifth, due to the exploratory nature of the study and limited prior data, structural equation modeling (SEM) was not applied to examine complex relationships among variables. Future research could collect multidimensional psychological data through pilot studies and build theory-based frameworks to support SEM analysis.

Based on the above findings and limitations, future research may consider the following directions. First, explore intelligent social support models by developing mobile platforms (e.g., WeChat mini programs or apps) for stoma patients, integrating remote nursing, peer communication, and health education to improve accessibility and continuity of support. Second, evaluate the effectiveness of policy interventions. Real-world studies (RWS) linked to the upcoming “Stoma Patient Care Initiative” in Guangxi may help assess policy outcomes and optimize implementation. Third, conduct cross-regional cultural comparison studies in collaboration with multiethnic provinces such as Yunnan and Guizhou to examine differences in social support and intervention strategies under diverse cultural contexts, and to develop more adaptable support models. Fourth, further build mediation models to explore the interactive mechanisms between physiological complications, self-efficacy, and social support, thereby refining theoretical frameworks and guiding the development of individualized interventions.

## Conclusion

**Figure 6:**
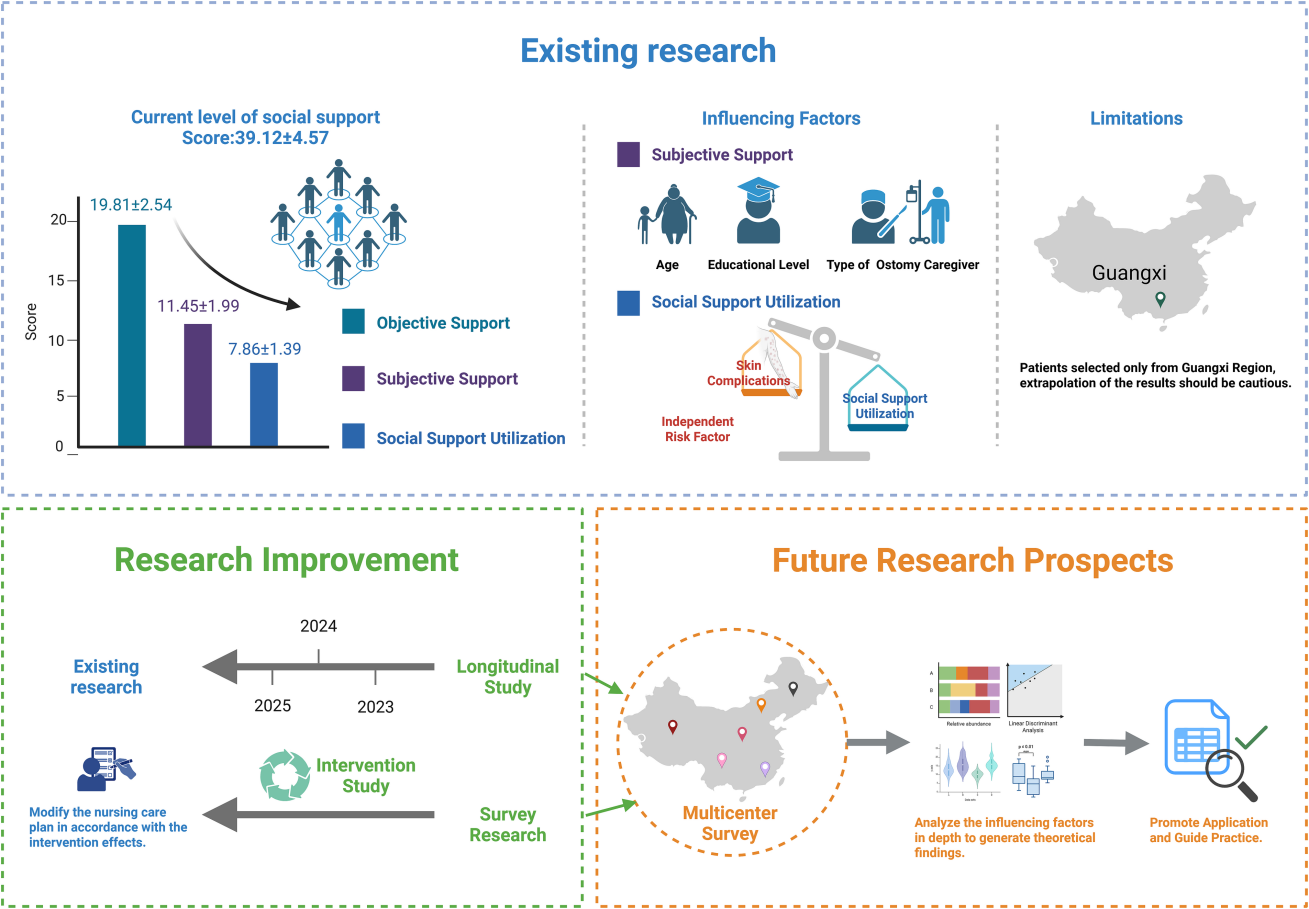
Current status and future directions of social support research in older adult patients with intestinal stomas.

This study systematically evaluated the level of social support and its primary influencing factors among older adult patients with intestinal stomas in the Guangxi region (Figure 6). The results revealed that the overall social support among patients was at a moderate level, with the type of primary stoma caregiver and the presence of peristomal skin complications identified as independent risk factors influencing social support. Further analysis indicated that differences in family caregiving roles and the effectiveness of managing peristomal skin complications were significantly associated with patients’ perception and utilization of social support. These findings not only enrich the regional evidence base in the field of psychosocial adaptation among stoma patients but also highlight the necessity of designing more targeted supportive interventions by taking into account cultural background, resource accessibility, and family structure, particularly in economically underdeveloped and multi-ethnic areas. Although this study had limitations in sample size and regional coverage, the results provide theoretical and practical references for future efforts to enhance social support levels among older adult patients with intestinal stomas through intelligent, culturally sensitive, and multi-level intervention approaches. Future research should incorporate longitudinal tracking designs and interventional studies to explore the dynamic mechanisms and optimization strategies of social support, thereby promoting continuous improvements in patients’ rehabilitation pathways and quality of life.

## Data Availability

The original contributions presented in the study are included in the article/supplementary material, further inquiries can be directed to the corresponding author.
